# Sonication of antibiotic-loaded cement spacers in a two-stage revision protocol for infected joint arthroplasty

**DOI:** 10.1186/1471-2474-14-193

**Published:** 2013-06-24

**Authors:** Massimo Mariconda, Tiziana Ascione, Giovanni Balato, Renato Rotondo, Francesco Smeraglia, Giovan Giuseppe Costa, Marco Conte

**Affiliations:** 1 Department of Orthopaedic Surgery, “Federico II” University, Policlinico Federico II, Via S. Pansini 5, bd. 12, 80128, Naples, Italy; 2 Department of Infectious Diseases, D. Cotugno Hospital, Naples, Italy; 3 Department of Orthopaedic Surgery, C.T.O. Hospital, Naples, Italy; 4 Laboratory of Microbiology, D. Cotugno Hospital, Naples, Italy

**Keywords:** Prosthesis-related infections, Sonication, Bone cements, Treatment outcome, Microbial biofilm

## Abstract

**Background:**

Culturing of the sonication fluid of removed implants has proven to be more sensitive than conventional periprosthetic tissue culture for the microbiological diagnosis of prosthetic joint infection. Since bacteria surviving on antibiotic-loaded cement spacers used in a two-stage exchange protocol for infected arthroplasties may cause the persistence of infection, in this study we asked whether the sonication also could be used to identify bacteria on antibiotic-loaded cement spacers removed at the second surgical stage during a two-stage exchange procedure to confirm whether or not the prosthetic joint infection had been eradicated.

**Methods:**

We cultured the sonication fluid of cement spacers that had been originally implanted in a two-stage exchange protocol in 21 patients (mean age, 66 years) affected by prosthetic joint infection (16 total knee prostheses and 5 hip prostheses). The cement spacers were vortexed for 30 seconds and then subjected to sonication (frequency 35–40 KHz). The resulting sonicate fluid was cultured for aerobic and anaerobic bacteria.

**Results:**

The sonication fluid culture of the removed spacer was positive in six patients (29%), with isolation of methicillin-sensible Staphylococcus Aureus (MSSA) in three cases, methicillin-resistant Staphylococcus Aureus (MRSA) in one case and Pseudomonas Aeruginosa in two cases. In three of these positive cases, the traditional culture of periprosthetic tissue was negative. Two patients with positive sonication culture of the spacer were successfully treated by early debridement of the revision prosthesis and systemic antibiotic therapy. In three patients a knee arthrodesis was planned and performed as the second surgical stage. In two of them the infection was caused by highly resistant Pseudomonas Aeruginosa. The other patient with a MSSA infection had been poorly compliant with the systemic antibiotic therapy due to her mental impairment. The patient originally affected by MRSA infection of his primary hip arthroplasty developed recurrent infection of his revision prosthesis and eventually underwent Girdlestone arthroplasty.

**Conclusions:**

The sonication culture can be used to discover any bacteria on the antibiotic-loaded cement spacer during a two-stage exchange protocol, thus permitting the adoption of timely treatment options, such as the early prosthetic debridment.

## Background

A two-stage exchange procedure is commonly used for treating a prosthetic joint infection (PJI). It entails removal of the prosthesis with debridement of all infected tissue followed by administration of antimicrobial therapy, and subsequent delayed reimplantation of a second prosthesis. The temporary implantation of an antibiotic-loaded cement spacer facilitates the ease and safeness of the revision because it permits us to preserve the joint space during the interim period and ensures local release of antibiotics. High rates of satisfactory outcomes in terms of infection eradication have been reported using the two-stage exchange procedure [1], but in some cases both the clinical picture and serum C-reactive protein (CRP) levels do not normalize over time even with the removal of the infected implant and the prolonged implantation of the antibiotic-loaded cement spacer [2]. In these cases, the presence of biofilm-forming pathogens may cause the infection to persist and the antibiotic-loaded cement itself can act as a biomaterial surface to which bacteria may preferentially adhere, grow and possibly even develop antibiotic resistance [3]. Ultrasound treatment may be used to dislodge biofilm bacteria from the surface of orthopaedic implants allowing significant recovery of microorganisms [4]. Culturing of the sonication fluid has proven to be more sensitive than conventional periprosthetic tissue culture for the microbiological diagnosis of PJI [5,6]. In this study we asked whether the sonication also could be used to identify bacteria on antibiotic-loaded cement spacers removed at the second surgical stage during a two-stage exchange procedure, a) to confirm whether or not the PJI had been eradicated and b) to compare the findings from conventional intra-operative tissue cultures with the findings after sonication of the cement spacer.

## Methods

From March 2009 to January 2011 we performed a two-stage exchange procedure in 21 consecutive patients, 10 women and 11 men, affected by PJI (16 total knee prostheses and 5 hip prostheses). Mean patient age was 66.1 years (range, 55–78 years). The diagnosis of suspected prosthetic infection was made on the basis of clinical signs and symptoms (reported pain, hyperthermia, swelling, redness), preoperative microbiological cultures (material from joint aspiration or sinus track), laboratory studies (leukocytosis, increased CRP), plain radiographs, and nuclear medicine findings (99 m-Tc HMPAO labeled leukocyte scan or 18-fluorodeoxyglucose positron emission tomography (FDG-PET) scan). To be included in this study, that was approved by the institutional review board of the Department of Surgery and Orthopaedics of Federico II University, Naples, Italy and is compliant with the Helsinki Declaration, all individuals gave their oral and written informed consent for the publication of individual clinical details. They met at least one of the following criteria to confirm the infection [7]: i) two or more cultures of joint aspirates or cultures of intraoperative specimens yielding the same microorganism; ii) purulence surrounding the prosthesis at the time of explantation; iii) acute inflammation consistent with infection during pathohistological examination; iv) a sinus tract that communicated with the prosthesis. Removal of the prosthesis with debridement of all infected tissue and placement of a temporary molded antibiotic-loaded cement spacer was performed in each case. All these spacers contained gentamicin plus clindamycin, but in three cases an extra 2 g vancomycin was added. At the time of the first surgical stage, five specimens of periprosthetic tissue were intraoperatively collected under sterile conditions and sent for microbiological and histopathological diagnosis. The prosthetic components were packed into sterile containers, covered with four-hundred millilitres of Ringer’s solution, and sent for sonication fluid analysis. The resection of prosthetic components was followed by intravenously administered empirical antibiotic treatment for one week until the cultures and susceptibility tests were available. Combination therapy including drugs showing activity against biofilms was used on an outpatient basis thereafter, according to the sensitivity profile of the cultured microorganisms. In 3 patients (14.3%) with negative pre- and intra-operative cultures and positive histology showing acute inflammation consistent with infection, an empirical combination antibiotic treatment including rifampin was given. The second surgical stage was performed after clinical and laboratory findings had normalized. Antibiotic therapy was discontinued at least two weeks before surgery. The time interval between the removal of the infected prosthesis and the reimplantation of the second prosthesis varied, depending on the clinical and laboratory response to antibiotics and the cultured microorganism (Table 1). At the time of the cement spacer removal, multiple specimens of periprosthetic tissue were collected and sent for microbiological and histopathological studies. The cement spacers were packed into sterile containers, covered with four hundred millilitres of Ringer’s solution, and sent for sonication. After the hospital discharge, patients were checked clinically, serologically, and radiographically at 2, 4, 12, and 24 weeks after the second surgery. Thereafter, clinical, serologic (CRP, leukocyte count), and radiographic checks were obtained every 6 months.

**Table 1:** Clinical, microbiological, and laboratory data for the 21 patients with prosthetic joint infection

**Patient No/Age, Y/Sex**	**Prosthesis**	**Medical condition**	**Pre-operative culture/ Microorganism**	**Pre-op CRP mg/dl**	**Intra-operative culture (i)**	**Sonication culture/Prosthesis**	**Pre-2**^**nd **^**stage CRP mg/dl**	**Interim Period/ Second stage surgery**	**Intra-operative culture (ii)**	**Sonication culture/Spacer**	**3**^**rd **^**stage surgery/Follow-Up/Final outcome**
1/74/M	TKA	none	joint aspiration fluid/PA	1.0	no growth	no growth	1.2	30 days/reimplantation	no growth	no growth	none/28 months/no recurrence
2/65/F	TKA	none	joint aspiration fluid/MSSA	1.8	MSSA	MSSA	1.2	30 days/reimplantation	no growth	MSSA	early debridement/25 months/no recurrence
3/70/M	TKA	none	culture of sinus tract/ PA	2.5	PA	PA	2.1	85 days/arthrodesis	PA	PA	none/23 months/no recurrence**/**bony fusion
4/68/M	TKA	none	joint aspiration fluid/CONS	2.8	CONS	CONS	0.4	87 days/reimplantation	no growth	no growth	none/25 months/no recurrence
5/71/F	TKA	none	joint aspiration fluid/no growth	2.3	CONS	CONS	0.3	90 days/reimplantation	no growth	no growth	none/24 months/no recurrence
6/70/M	TKA	none	joint aspiration fluid/no growth	30.0	CONS	CONS	0.2	90 days/reimplantation	no growth	no growth	none/28 months/no recurrence
7/71/M	TKA	none	joint aspiration fluid/no growth	3.1	CONS	CONS	0.3	92 days/reimplantation	no growth	no growth	none/27 months/no recurrence
8/78/M	TKA	none	culture of sinus tract/ PA	3.5	PA	PA	1.0	92 days/ arthrodesis	PA	PA	none/25 months/no recurrence**/**fibrous union
9/72/M	TKA	none	joint aspiration fluid/CONS	4.1	CONS	CONS	0.4	95 days/reimplantation	no growth	no growth	none/27 months/no recurrence
10/55/F	THA	none	joint aspiration fluid/no growth	3.5	MSSA	MSSA	0.3	100 days/reimplantation	no growth	MSSA	early debridement/20 months/no recurrence
11/64/M	TKA	none	joint aspiration fluid/MSSA	1.8	MSSA	MSSA	0.3	120 days/reimplantation	no growth	no growth	none/26 months/no recurrence
12/55/F	TKA	diabetes	joint aspiration fluid/no growth	0.3	MSSA	CONS	0.3	120 days/reimplantation	no growth	no growth	none/27 months/no recurrence
13/62/F	THA	diabetes	none	15.0	CONS	CONS	0.3	125 days/reimplantation	no growth	no growth	none/23 months/no recurrence
14/64/F	TKA	none	joint aspiration fluid/no growth	1.9	no growth	no growth	0.3	150 days/reimplantation	no growth	no growth	none/23 months/no recurrence
15/63/M	TKA	none	joint aspiration fluid/MSSA	2.0	MSSA	none	1.7	150 days/reimplantation	no growth	no growth	none/26 months/no recurrence
16/68/M	THA	psoriasis	culture of sinus tract/MRSA	4.4	MRSA	MRSA	0.4	180 days/reimplantation	no growth	MRSA	Girdlestone arthroplasty 18 months/no recurrence
17/58/F	TKA	schizophrenia	culture of sinus tract/ MSSA	0.5	CONS	MSSA	0.7	180 days/arthrodesis	MSSA	MSSA	none/24 months/no recurrence**/**bony fusion
18/60/F	THA	none	culture of sinus tract/MSSA	30.0	MSSA	MSSA	0.3	180 days/reimplantation	no growth	no growth	none/26 months/no recurrence
19/66/M	THA	none	none	5.8	CONS	CONS	0.4	180 days/reimplantation	no growth	no growth	none/22 months/no recurrence
20/69/F	TKA	none	joint aspiration fluid/CONS	2.0	CONS	CONS	0.5	210 days/reimplantation	no growth	no growth	none/25 months/no recurrence
21/64/F	TKA	none	joint aspiration fluid/no growth	1.8	no growth	no growth	0.3	240 days/reimplantation	no growth	no growth	none/29 months/no recurrence

Abbreviations: *CRP* C-reactive protein, *TKA* total knee arthroplasty, *THA* total hip arthroplasty, *MSSA* methicillin-sensible *Staphylococcus aureus*, *MRSA* methicillin-resistant *Staphylococcus aureus*, *CONS* coagulase-negative *Staphylococcus*, *PAPseudomonas Aeruginosa.*

### Culture of tissue specimens

Tissue specimens were homogenized in 3 ml of brain-heart-infusion broth for 1 min and the homogenate was inoculated in aliquots of 0.5 ml. Aerobic and aerobic sheep-blood agar were incubated at 35°C to 37°C in 5% to 7% carbon dioxide aerobically and anaerobically for 5 days and 7 days, respectively.

### Sonication protocol

The container was vortexed for 30 seconds using a Vortex-Genie (Scientific Industries, Inc, Bohemia, NY, USA) and then subjected to sonication (frequency 35–40 KHz) in an Aquasonic Model 750 T ultrasound bath (VWR Scientific Products, Radnor, PA, USA) for 5 minutes, followed by additional vortexing for 30 seconds. The resulting sonicate fluid was plated in 500 μl aliquots onto aerobic Columbia sheep blood agar plates for 5 days and anaerobic Shaedler sheep blood agar for 7 days. Microorganisms were enumerated and classified by routine microbiological techniques. A total of 200 ml of sonicate fluid was centrifuged at 2600 rpm for 15 minutes and the sediment was gram stained. All bacteria were counted and identified by standard methods (VITEK Biomerieux, Bagno a Ripoli (FI), Italy).

### Statistical analysis

Continuous variables are expressed as the median and interquartile range (IQR). Categorical variables were analyzed with a chi-squared test or Fisher’s exact test. P < 0.05 was considered statistically significant. SPSS software (SPSS Inc., Chicago, Illinois, USA) was used for the database and statistics.

## Results

Table 1 presents the clinical and microbiological results of the patients who underwent a two-stage procedure to treat their PJI. The median length of the interim period between the two surgical stages was 120 days (IQR 90 to 180) and the median length of follow-up after last surgical procedure was 25 months (IQR 23 to 27). At the time of the second surgical stage, 18 patients underwent a reimplantation procedure and the remaining 3 patients received a knee arthrodesis. The sonication fluid culture of the removed spacer was positive in six cases (29%) and the traditional culture of the periprosthetic tissue was positive in only three of these. In all cases with positive sonicate fluid culture of the removed spacer, the same pathogen that had caused the initial infection grew in the sonication culture of the cement spacer. In detail (Table 1), we detected three methicillin-sensible *Staphylococcus aureus* (MSSA), one methicillin-resistant *Staphylococcus aureus* (MRSA), and two *Pseudomonas Aeruginosa* infections. Two patients with MSSA infection (Table 1 – patients n. 2 and n. 10) of their spacer were successfully treated by aggressive debridement of the prosthetic joint within 30 days of prosthesis implantation and systemic antibiotic therapy tailored to the sensitivity of the cultures for 3 months. In three patients a knee arthrodesis was planned and performed as the second surgical stage. Two of them with highly resistant *Pseudomonas Aeruginosa* PJI (Table 1 – patients n. 3 and n. 8) did not respond to antibiotic therapy and presented wound dehiscence and opening of a sinus track. The third patient who received the knee arthrodesis was affected by MSSA infection (Table 1 – patient n. 17) and had been poorly compliant with the systemic antibiotic therapy due to her mental impairment. Both the traditional intraoperative culture at the time of the second surgical stage and the sonication culture of the cement spacer in these three patients were positive. The patient originally affected by MRSA infection of his primary total hip arthroplasty (THA)(Table 1 – patient n. 16) underwent removal of the infected prosthesis and a gentamicin and clindamycin-loaded cement spacer containing extra vancomycin was implanted. This patient firmly required to undergo implantation of a new THA at the second surgical stage and because of the complete normalization of clinical, laboratory, and nuclear medicine findings, a reimplantation was performed 180 days after the removal of the primary prosthesis. Unexpectedly, MRSA was isolated by the sonication culture of his cement spacer. This patient developed recurrent infection of his revision THA and eventually underwent Girdlestone arthroplasty.

## Discussion

Two-stage exchange is the standard in the treatment of PJI. High frequency of success has been reported with this strategy of management, with a rate of eradication of both total knee arthroplasty [8] and THA [9] infection of more than 80 percent. Although not evaluated in randomized controlled trials, the application of a temporary antibiotic-loaded bone cement spacer in the interim period before revision surgery is largely used in the two-stage exchange protocol because it enables preservation of the joint space after removal of the primary joint arthroplasty and ensures high local concentrations of antibiotics that can help to eradicate the infection. Despite the adoption of the two-stage exchange protocol, there are cases of persisting PJI where the cement spacer itself may act as a biomaterial surface that facilitates survival of microorganisms [3,10]. Hence, it is of paramount importance to actively look for biofilm bacteria on the removed cement spacer to determine whether the infection has been cleared. Nevertheless, the limited sensitivity and specificity of standard microbiological culture techniques may limit their ability to detect the adherent bacteria responsible for PJI [11,12]. The application of ultrasound before culturing may disrupt the biofilm from the surface of orthopaedic implants and free the bacteria, enhancing the sensitivity of traditional cultures [5,6]. In the present study we used the sonication fluid culture of cement spacers to confirm the eradication of the infection obtained by a two-stage exchange protocol. We are aware of only one recent study that applied sonication culture to antibiotic-loaded spacers to identify bacterial growth on the cement spacer surface [13]. This study reported a 14.5% rate of positive sonication fluid cultures of the removed spacer and showed that an infection of the cement spacer is associated with poor clinical outcome. The sonicate fluid culture of the removed spacer in our study was positive in six cases (29%). In three of these positive cases, the traditional culture of periprosthetic tissue failed to identify the pathogen. One notable difference with our results is that most sonication fluid cultures of the spacer in the above cited study were discordant with the culture of both the prosthesis (first-stage surgery) and peri-prosthetic tissue specimens (second-stage surgery). If a positive culture is obtained from the sonication process that reveals the same bacteria that caused the initial PJI as in the present study, it should be aggressively treated. The idea would be that in these cases a one stage exchange had been performed and a regimen of prolonged antibiotic therapy should be adopted.

Our results agree with the findings of other authors who reported MSSA and MRSA on gentamicin-and gentamicin-vancomycin-loaded cement beads, respectively, using traditional cultures [3,14]. For patients in whom pathogens are isolated on the cement spacer the outcome should be guarded, particularly when the infection is sustained by highly resistant microorganisms. Indeed, the patient with MRSA in this study developed a recurrent infection even though a cement spacer containing vancomycin had been used. The emergence of resistance in bacterial strains is a concern about the long-term exposure to antibiotics at the site of a previous infection [15]. In the case of MRSA infections, the prolonged implantation of gentamicin/vancomycin-loaded spacers may be associated with bacterial survival, and hence with the persistence of infection [14], particularly when an insufficient debridement has been carried out at the time of explantation of the primary prosthesis. Other studies have reported high failure rates after two-stage reimplantation when the infection is sustained by methicillin-resistant staphylococci [16-19]. In cases of PJI sustained by highly virulent microorganisms for which there is limited medical therapy, the arthrodesis or resection arthroplasty may be a viable option as the second stage surgery [20]. We performed a knee arthrodesis as the second surgical stage in three patients who either failed to respond or were poorly compliant to the antibiotic therapy. This proved to have been a fair therapeutic choice because of the culture of both the sonication fluid and the periprosthetic tissue on samples collected at the time of the second surgical stage which showed persistence of the infection in these three patients.

Some reservations must be stated with regard to the interpretation of our results. One doubt emerging from the present study is the practical usefulness of a diagnostic test on the cement spacer carried out after the reimplantation has already been performed. Even though one previous *in vivo* study showed bacterial biofilm adhesion to orthopaedic metals in the first 48 hours after implantation [21], the reinfection of the reimplanted prosthesis should be regarded and treated as an early infection with a retention strategy, because bacterial adhesion to the new implant and the biofilm formation have not yet been fully realized. Indeed, our results and those of previous studies [22-24] have demonstrated that patients with a short-term infection with a known pathogen and a stable implant may be successfully treated by early debridement, with retention of the prosthesis and the use of a standardized regimen of antimicrobial treatment. The major limitation of this study is the small sample size, which implies low statistical power. Moreover, although the mean duration of implantation of the cement spacer between the two surgical procedures in a two-stage protocol is highly variable in the literature, in our study it was long and it could be questioned that the long time that had elapsed between the two surgical stages may have affected the bactericidal power of the cement spacer and caused the growth of bacteria on its surface [1]. Actually, the ability of cement spacers to ensure long-lasting bactericidal levels of antibiotics is controversial. After one day, subinhibitory concentrations of antibiotics on *Staphylococcus aureus* strains from different cements have been detected *in vitro*[25] and a declining trend over time in the antibiotic release from cement spacers has been reported *in vivo*[26,27]. Conversely, other *in vivo* studies have shown that antibiotics released from the spacers result in long lasting bactericidal concentrations in the peri-prosthetic tissue [15,28]. Individual variations in the local perfusion, the different characteristics of the bone cement (porosity, roughness, total surface area), and the method and doses adopted to prepare the antibiotic-impregnated cement may lead to a variable in vivo concentration and bioactivity of the antibiotics released from the cement spacers and help to explain the discrepancy between the published data [15,27]. However, independent of the bactericidal power of the cement spacer, positive clinical results have been reported with long cycles of combined oral antibiotics plus a delayed reimplantation in the two-stage exchange protocol, even in infections sustained by highly resistant bacteria [29].

## Conclusions

Bacteria may survive on antibiotic-loaded cement spacers, irrespective of the fact that they may have been preoperatively susceptible to the antibiotics included in the spacer. This might eventually result in a clinical reinfection. The sonication culture is a complementary method that can help to discover the possible persistence of microorganisms on an antibiotic-loaded cement spacer during a two-stage exchange protocol. Its use may help to confirm whether an infection has been definitely cleared, or whether further therapeutic options, such as the early debridment of the revision prosthesis, are necessary. Our results emphasize the importance of systemic antibiotics, given as a complement to the spacer implantation.

## Abbreviations

PJI: Prosthetic joint infection; CRP: C-reactive protein; IQR: Interquartile range; MSSA: Methicillin-sensible Staphylococcus aureus; MRSA: Methicillin-resistant Staphylococcus aureus; THA: Total hip arthroplasty.

## Competing interest

The authors declare that they have no competing interests.

## Authors’ contribution

MM: Conception and design, analysis of data, writing the manuscript, supervision. TA: Conception and design, analysis of data, critical revision of the manuscript. GB: Acquisition, analysis and interpretation of data, preparation of the manuscript. RR: Acquisition, analysis, and interpretation of data, critical revision of the manuscript. GGC and FS: Acquisition, analysis, and interpretation of data, preparation of the manuscript. MC: Microbiological analysis, sonication, analysis of data. All authors read and approved the final manuscript.

## Pre-publication history

The pre-publication history for this paper can be accessed here:

http://www.biomedcentral.com/1471-2474/14/193/prepub
